# Gene Expression Profiling of Soft and Firm Atlantic Salmon Fillet

**DOI:** 10.1371/journal.pone.0039219

**Published:** 2012-06-20

**Authors:** Thomas Larsson, Turid Mørkøre, Kari Kolstad, Tone-Kari Østbye, Sergey Afanasyev, Aleksei Krasnov

**Affiliations:** 1 Nofima, Ås, Norway; 2 Department of Animal and Aquacultural Sciences, Norwegian University of Life Sciences, Ås, Norway; 3 Sechenov Institute of Evolutionary Physiology and Biochemistry, St Petersburg, Russia; Pennsylvania State University, United States of America

## Abstract

Texture of salmon fillets is an important quality trait for consumer acceptance as well as for the suitability for processing. In the present work we measured fillet firmness in a population of farmed Atlantic salmon with known pedigree and investigated the relationship between this trait and gene expression. Transcriptomic analyses performed with a 21 K oligonucleotide microarray revealed strong correlations between firmness and a large number of genes. Highly similar expression profiles were observed in several functional groups. Positive regression was found between firmness and genes encoding proteasome components (41 genes) and mitochondrial proteins (129 genes), proteins involved in stress responses (12 genes), and lipid metabolism (30 genes). Coefficients of determination (R^2^) were in the range of 0.64–0.74. A weaker though highly significant negative regression was seen in sugar metabolism (26 genes, R^2^ = 0.66) and myofiber proteins (42 genes, R^2^ = 0.54). Among individual genes that showed a strong association with firmness, there were extracellular matrix proteins (negative correlation), immune genes, and intracellular proteases (positive correlation). Several genes can be regarded as candidate markers of flesh quality (*coiled-coil transcriptional coactivator b*, *AMP deaminase 3*, and *oligopeptide transporter 15*) though their functional roles are unclear. To conclude, fillet firmness of Atlantic salmon depends largely on metabolic properties of the skeletal muscle; where aerobic metabolism using lipids as fuel, and the rapid removal of damaged proteins, appear to play a major role.

## Introduction

The Norwegian salmon industry aims to increase the production of value-added products in order to improve profitability [1]. Understanding the biological differences in fish of varying fillet quality is thus an area where knowledge is needed to enable targeted actions. Fillet firmness of farmed salmon is an important quality trait for consumer acceptance [2], [3], and poor firmness results in downgrading during secondary processing [4]. Texture is a multifaceted characteristic influenced by both ante- and post-mortem factors [5]. Ante- mortem factors affecting fillet quality characteristics such as texture include genetic background [6], [7], feed and feeding [8], environmental factors [9] and health status [10], [11]. Given the highly complex nature of this trait and limited knowledge, it is expedient to use high-throughput screening methods, which may help to distinguish and evaluate the effects of various factors.

Microarray technology presents a powerful tool for revealing expression patterns and genes associated with phenotypic characteristics [12]. By determination of expression levels of thousands of genes simultaneously in muscle tissue, it may be possible to reveal global gene expression patterns and to identify genes or groups of genes associated with texture variations. Microarray has been used to associate differential gene expression with meat quality of porcine muscle [13]. Salem et al. [14] studied gene expression in atrophying rainbow trout muscle, but no studies have used microarray to examine molecular causes to variations in fillet firmness of salmon muscle. The aim of the present work was to elucidate the transcriptional profile of farmed salmon muscle with varying firmness. The study applied multiple gene expression profiling in white skeletal muscle using a recently developed Atlantic salmon oligonucleotide microarray supplemented with bioinformatic system STARS [15]. As part of a larger study, we took advantage of fish material from a breeding program, which provided access to salmon with known pedigrees and that were reared under standard conditions. Sixteen individuals were selected from a large number of fish based on instrumental texture measurements; this group covered the whole range of meat firmness observed in salmon produced by the Norwegian industry [16]. This study presents for the first time the relationship between individual genes and functional groups, and the texture of salmon fillets. The results suggest that this trait is associated largely with intracellular metabolic processes.

## Materials and Methods

### Ethics Statement

Rearing and slaughtering were conducted at Nofima Research station (Averøy, Norway) which is approved by Norwegian Animal Research Authority (NARA), and stunning and sampling of fish were performed in accordance with the Norwegian Animal Welfare act. Fish were treated as production fish up to the point of tissue sampling which was done only after fish were put to death. Hence, no NARA approval was required according to Dr. G Baeverfjord (Nofima), appointed by NARA.

### Fish Material and Sampling

A resource population of Atlantic salmon (*Salmo salar* L.) with known pedigree (98 full- and half-sib families, n = 944 individuals) was provided by the breeding company SalmoBreed AS, Norway. Fish were transferred to seawater in May 2007 as 1+ smolts and reared in cages (400 m^3^) at Nofima’s research station in Averøy, Norway. All fish were sacrificed in September 2008 by percussive stunning and bled in fresh seawater after cutting the left gill arches. Fat content was predicted on whole fish by NIR [17] and weight and length were recorded. The salmon were then hand-filleted and white muscle anterior to the dorsal fin and immediately above the lateral line taken and frozen immediately in liquid nitrogen and stored at −80°C until required for analyses of gene expression. The fillets were packed in polystyrene boxes with ice and transported to Nofima (Ås, Norway), where they were analyzed for texture four days post-mortem. The average body weight of the salmon was 3.5 kg (SD 0.9 kg) for the total population. Based on measurements of fillet firmness, 16 fish with normal exterior appearance (superior quality grade), average condition factor (1.2±0.0) and fat content (19% ±0.6), were selected for analyses of gene expression.

### Texture

Texture analyses were performed instrumentally (TA-XT2, Stable Micro Systems Ltd., Surrey, England) by pressing a flat-ended cylinder (12.5 mm diameter, type P/0.5) into the fillet below the dorsal fin perpendicular to the muscle fibers at 1 mm/sec until it reached 60% of the fillet height. The force (N) required to puncture the fillet surface (termed firmness) was registered from the resulting time-force graph.

### Calculation of Heritability

Genetic and environmental variances and co-variances were estimated using a general mixed linear analysis based on an animal model using the DMU software [18]. The model included the fixed effects of degree of maturation and age, and the random effects of animal and common environment of family. Pedigree information was included in the analysis. The heritability was calculated as the ratio between additive genetic effects and the sum of all genetic and environmental effects.

### Gene Expression Analyses

#### Isolation of RNA

Gene expression in each of the 16 selected fish was analyzed by real time quantitative RT-PCR (qPCR) [19] and by microarray. RNA was isolated from white muscle using the PureLink RNA Mini Kit (Invitrogen Corporation, Carlsbad, CA). Tissue was homogenized in TRIzol in a Precellys 24 (Bertin technologies). RNA integrity was assessed with Agilent 2100 Bioanalyzer (Agilent Technologies).

#### Real-time quantitative RT PCR

For qPCR, four reference genes were tested (*elongation factor 1a, eukaryotic translation initiation factor 3, RNA polymerase 2* and *β-actin*) and *elongation factor 1a* proved the most stable and was used as reference gene [20]. The primers are in Table 1.

**Table 1:** Primers used for qPCR.

Target gene	Forward primer (5′–3′)	Reverse primer (5′–3′)	Genbank accession no.
*carnitine palmitoyltransferase I*	GTACCAGCCCCGATGCCTTCAT	TCTCTGTGCGACCCTCTCGGAA	AM230810
*elongation factor 1a*	CACCACCGGCCATCTGATCTACAA	TCAGCAGCCTCCTTCTCGAACTTC	AF321836
*eukaryotic translation initiation factor 3*	CAGGATGTTGTTGCTGGATGGG	ACCCAACTGGGCAGGTCAAGA	DW542195
*malate dehydrogenase*	AGACGTCCACCACTCCAAGGTCAA	TTAACAGGGTCGAAGCAGGCCA	BT045320
*myogenin*	ATTGAGAGGCTGCAGGCACTTG	GTGCGGTAGTGTAAGCCCTGTGTT	DQ294029
*pparα*	TCCTGGTGGCCTACGGATC	CGTTGAATTTCATGGCGAACT	DQ294237
*pparβ*	GAGACGGTCAGGGAGCTCAC	CCAGCAACCCGTCCTTGTT	AJ416953
*RNA polymerase II*	TAACGCCTGCCTCTTCACGTTGA	ATGAGGGACCTTGTAGCCAGCAA	CA049789
*β-actin*	ACATCAAGGAGAAGCTGTGC	GACAACGGAACCTCTCGTTA	AF012125
*creatine kinase m3*	GGAAACTGATCCAGGATGTTGCA	CGCTTAGAGTAAACTGATGCTCGCTC	DN165248

#### Microarray platform

Design of Nofima’s Atlantic salmon oligonucleotide microarrays (ONM) and bioinformatic system STARS was reported in [15]. In brief, mRNA sequences retrieved from public databases (Unigene, Genbank and The Gene Indices) were identified by blastx comparison with proteins of salmonid species (Genbank), zebrafish and human (Refseq and Uniprot). Atlantic salmon genes were linked to the functional classes (Gene Ontology – GO) and pathways (KEGG) by closest human and zebrafish proteins found with aid of blastx. For several groups annotation with GO and KEGG appeared insufficient. Genes that were identified only by salmonid proteins with no match to known human and zebrafish proteins remained without annotations. Mining of databases and scientific publications revealed gaps and inaccuracies for many annotated genes. Therefore custom annotation of Atlantic salmon sequences was implemented in STARS. The 60-mer oligonucleotide probes to unique protein coding sequences were designed by earray (Agilent Technologies).

#### Microarray hybridization and primary processing of data

The microarrays were fabricated by Agilent Technologies in the 44 K×4 format, each probe was printed in duplicate. All reagents and equipment were purchased from the same source. Dual-label hybridizations were carried out using a pool of all 16 samples as a common technical reference. RNA labeling and amplification was performed with Low Input Quick Amp Labeling Kits, Two-Color and RNA Spike-In Kits, Two-Color using 200 ng of total RNA per reaction. For fragmentation of the labeled RNA, Gene Expression Hybridization Kits were used. Labeled RNA was hybridized for 17 hours in an oven at 65°C and rotation speed of 10 rounds per minute. Arrays were washed for one minute with Gene Expression Wash Buffer I at room temperature, and one minute with Gene Expression Wash Buffer II at 37°C. Slides were scanned using GenePix Personal 4100A scanner (Molecular Devices, Sunnyvale, CA, USA) at 5 µm. The GenePix Pro software (version 6.1) was used for spot-grid alignment, feature extraction and assessment of spot quality. Unless specified otherwise, subsequent data analyses were carried out with STARS. Files generated by GenePix (gpr) were transferred in the relational database (MySQL). Low quality spots were filtered by flags assigned with GenePix. This simple and reliable criterion was chosen based on comparison of several methods [15]. Log_2_-Expression Ratios (ER) were calculated and Lowess normalization was performed [21]. Analyses continued with 12650 genes that passed quality control in at least 12 of 16 samples (Table S1).

#### Data analyses, statistics

Statistical analyses of microarray results were performed using Statistica and MS Excel. Correlation with firmness was analyzed and coefficient of linear regression (S – slope) was determined for each gene. The P-values of Pearson r were corrected for False Discovery Rate – FDR [22] and Q-values were calculated. The differentially expressed genes (DEG) were selected at the cut-off values: Pearson r > |0.6|, slope > |0.094|, which corresponded to 2.5-fold difference within the range and Q <0.05; 579 genes met these criteria (Table S2). Data analyses that included annotations of genes were performed with STARS. A search for enriched GO classes and KEGG pathways in the list of DEG was performed by counting of genes among DEG and all genes that passed quality control. Enrichment was assessed with Yates’ corrected chi square test (P<0.05); the terms with less than five genes were not taken into consideration. Relationship with firmness was assessed for the functional groups that included genes with highly correlated expression profiles. The mean log_2_-ER values were calculated and linear regression analysis was performed using Statistica. Multiple regression analysis was applied to evaluate the input of different groups. The data were deposited in NCBI's Gene Expression Omnibus (GEO Series accession number: GSE36475).

## Results

### Texture

For the total material, the fillet firmness presented a normal distribution with an average of 11.2 N (range: 5.5–20.9 N, SD 2.1 N). The heritability of texture was calculated to be 0.16. Muscle firmness of the sixteen selected individuals was 12.3 N on average, ranging from 6.8 to 20.9 N.

### qPCR and Microarray

**Figure 1:**
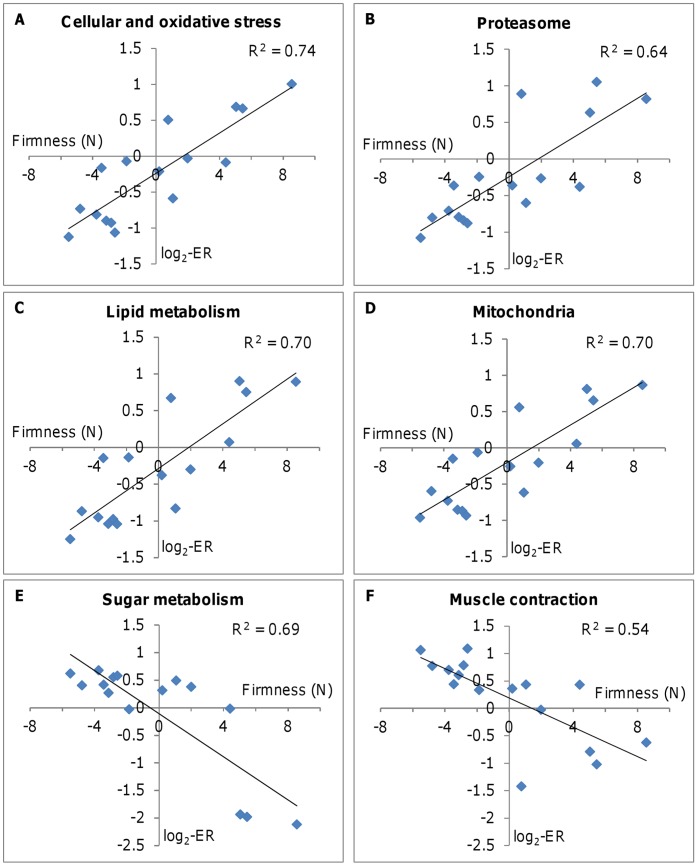
Relationship between fillet firmness and expression of genes from different functional groups in salmon skeletal muscle (n = 16 fish). Data are mean centered values of firmness (X-axis) and mean log_2_-ER (Y-axis), and R^2^ is the coefficient of determination. A: responses to protein and oxidative stress (11 genes); B: proteosomal degradation of proteins (41 genes); C: lipid and steroid metabolism (30 genes); D: mitochondrial proteins (129 genes); E: metabolism of sugars (26 genes); F: muscle contraction (42 genes).

A search for the enriched functional classes of GO and KEGG pathways was performed for initial screening of thematic associations of genes that showed high correlation with fillet firmness (Table 2). The major part of the enriched terms was related to metabolism of amino acids, sugars, lipids and proteins, and metabolically active cellular compartments (mitochondria, peroxisomes and proteasomes). The enrichment analysis did not take into account the direction of gene expression differences and several groups (e.g. those related to amino acids metabolism) included genes that showed either positive or negative correlation with firmness. The functional classes and pathways were compared by the counts of genes; the quantitative character and the strength of relationship were not assessed. Finally, semi-automatic annotation by GO and KEGG underestimated the numbers of genes corresponding to several functional groups; therefore, data analysis was continued with the aid of custom annotations provided by STARS. Several groups showed a strong association with the fillet firmness, which was well described with linear regression (P<0.001). Data are represented further as scatter plots of mean log_2_-ER values with regression lines (Fig. 1), the gene composition of groups and expression data are in Table S3.

Highly correlated expression profiles and a strong positive correlation with firmness was seen for genes involved in responses to oxidative and protein stress (Fig. 1A). This group includes chaperones, cognates and protein-modifying enzymes that assist protein folding (e.g peptidyl-prolyl cis-trans isomerise). Thioredoxin and peroxiredoxins are proteins that regulate the redox status of cells [23]. A comparable trend was observed in a large group of genes encoding enzymes of the ubiquitin pathway and components of the proteasome that are involved in intracellular degradation of proteins (Fig. 1B).

Positive correlations with firmness were also shown for genes involved in different aspects of lipid metabolism (Fig. 1C): plasma transport and cell absorption, biosynthesis and oxidation. Carnitine is important in transporting long-chain fatty acids from the cytoplasm to the mitochondria, where they are used as a source of energy via beta-oxidation [24]. Several genes are involved in metabolism of carnitine, and the group of genes related to the carnitine metabolism (5 genes, data not shown) correlated positively with firmness (r = 0.79). The greatest positive correlation was shown by *β-carotene oxygenase*. Similar expression profiles were seen in genes encoding proteins in mitochondria (Fig. 1D) and peroxisomes, which are major sites of fatty acids metabolism. A large number of the differentially expressed mitochondrial genes are involved in electron transport and oxidative phosphorylation (46 genes), protein biosynthesis and folding (21 genes), lipid metabolism (9 genes) and the citrate cycle (TCA, 8 genes). The remaining genes take part in biosynthesis of cofactors and other metabolic pathways, trafficking and transport, and division of mitochondria and peroxisomes.

The negative correlations shown by genes for enzymes of sugar metabolism (absorption, glycogen degradation, glycolysis) and myofiber proteins (Fig. 1E, F) were weaker since differences were manifested mainly in the individuals with highest firmness. Two myofiber components did not follow the common trend. Filamin is a large actin cross-linking protein [25] while myozenin binds to Z-disk proteins and directs calcineurin signaling to the sarcomers [26]. These genes had higher expression in firm fillets. Multiple regression suggested equal by strength relationship between firmness and the functional groups (data not shown).

**Figure 2:**
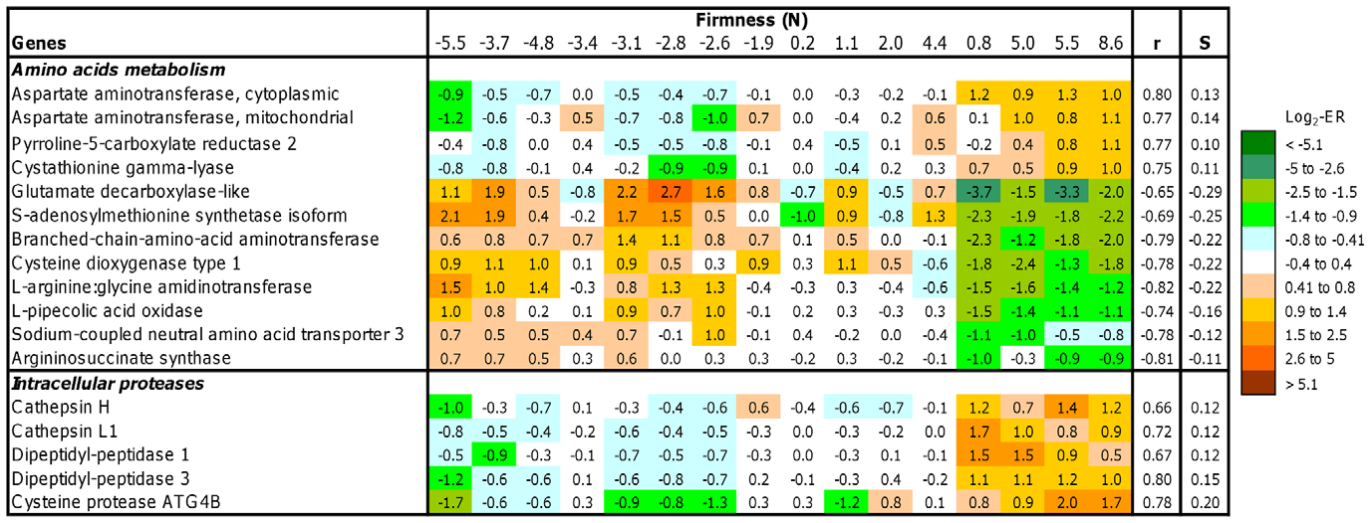
Expression of genes associated with amino acid metabolism and intracellular proteases in skeletal muscle of salmon with varying firmness (n = 16 fish). Data are log_2_-ER and are highlighted with a color key. r =  Pearson correlation coefficient, S =  coefficient of linear regression (S – slope).

The number of genes for intracellular proteases was relatively small among DEG and their expression was higher in fish with firm fillets (Fig. 2). Such expression profiles were seen in transcripts of *cathepsins H* and *L*. Relationship between firmness and amino acids metabolism was suggested by results of enrichment analysis (Table 2). Both negative and positive correlations were observed for genes involved in amino acids transport, biosynthesis and degradation of aspartate and glutamate, valine and leucine (*branched chain amino acid aminotransferase*), arginine and proline (*argininosuccinate synthase*, *pyrroline-5-carboxylate reductase* and *L-arginine:glycine amidinotransferase*), cysteine and methionine (*S-adenosylmethionine synthetases*, *cystathionine gamma-lyase* and *cysteine dioxygenase*).

**Table 2:** Enrichment of GO classes and KEGG pathways in the lists of DEG.

*Functional group, pathway*	*Vocabulary*	*Genes*		*Counrts* 1	*P-value*
Cysteine and methionine metabolism	KEGG	8	/	54	0.0032
Lysine degradation	KEGG	8	/	53	0.0027
Alanine metabolism	KEGG	6	/	39	0.0105
Tryptophan metabolism	KEGG	6	/	43	0.0198
Mitochondrion	GO	138	/	919	0
Oxidative phosphorylation	KEGG	36	/	158	0
Citrate cycle (TCA cycle)	KEGG	9	/	62	0.0019
Glycolysis/Gluconeogenesis	KEGG	28	/	120	0
Pentose phosphate pathway	KEGG	14	/	59	0
Lipid metabolic process	GO	21	/	221	0.0022
Fatty acid metabolic process	GO	17	/	79	0
Peroxisome	GO	13	/	102	0.0008
Proteasome	KEGG	41	/	79	0
Ribosome	GO	18	/	196	0.0075
Endopeptidase activity	GO	16	/	31	0
Muscle contraction	KEGG	14	/	102	0.0002
Adipocytokine signaling pathway	KEGG	8	/	70	0.0248
Insulin signaling pathway	KEGG	13	/	133	0.0146
PPAR signaling pathway	KEGG	11	/	68	0.0001
Regulation of Rho protein signal transduction	GO	19	/	93	0
Antigen processing and presentation	KEGG	7	/	60	0.0343
Extracellular space	GO	19	/	234	0.0248
Heparin binding	GO	9	/	65	0.003
Mitosis	GO	20	/	210	0.0028

1 Numbers of genes corresponding to term in the list of DEG/among all genes that passed quality control.

**Figure 3:**
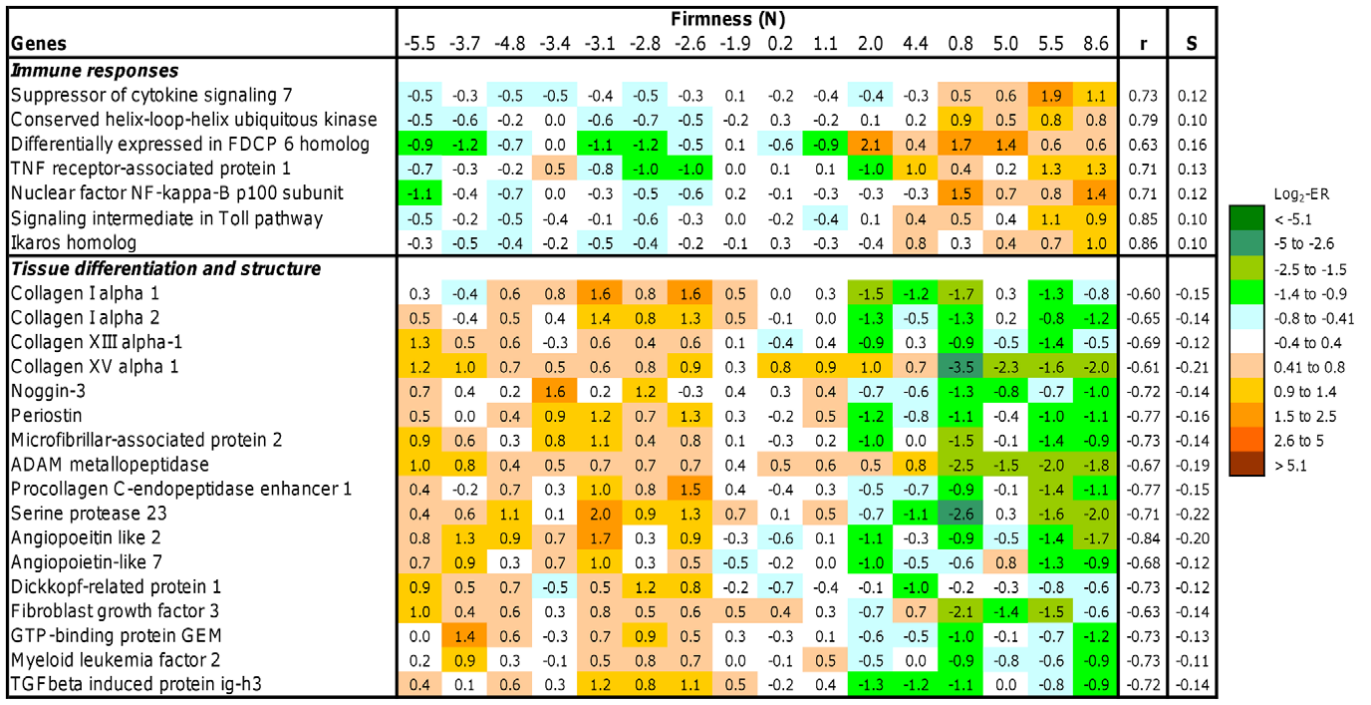
Expression of genes associated with immune response, tissue structure and remodeling in skeletal muscle of salmon with varying firmness (n = 16 fish). Data are log_2_-ER and are highlighted with a color key. r =  Pearson correlation coefficient, S =  coefficient of linear regression (S – slope).

Several immune genes showed positive correlation with firmness (Fig. 3) while an opposite tendency was observed in genes encoding proteins of the extracellular matrix – ECM (collagens, microfibrillar-associated protein 2 and periostin) and proteins involved in deposition and turnover of the ECM (ADAM metallopeptidase and procollagen C-endopeptidase enhancer [27]) and differentiation of connective tissue (TGFβ induced protein ig-h3 [28], angiopoeitin like 3 and 7, FGF3 and noggin [29]).

**Figure 4:**
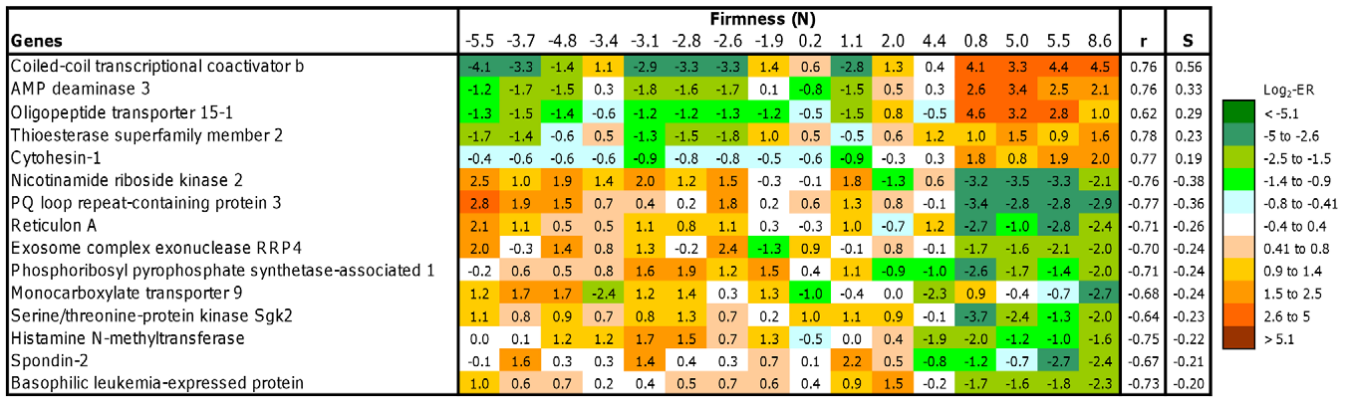
Expression of individual genes (candidate markers) correlating with firmness of salmon skeletal muscle (n = 16 fish). Data are log_2_-ER and are highlighted with a color key. r =  Pearson correlation coefficient, S =  coefficient of linear regression (S – slope).

Finally, a strong relationship with firmness was shown by a number of genes whose roles are unknown or whose association with the trait can hardly be explained based on their functions (Fig. 4). Highest S-values were seen in three probes to non-overlapping parts of the *coiled-coil coactivator* (CoCoA), which probably corresponded to the same transcript. CoCoA is involved in transcriptional activation of target genes by nuclear receptors including the aryl hydrocarbon receptor (a major regulator of xenobiotic metabolism), as well as target genes of the Wnt signaling pathway that plays an important part in differentiation of various cells [30]. AMP deaminase catalyses deamination of adenosine monophosphate while oligopeptide transporter 15 is involved in proton-coupled intake of oligopeptides of two to four amino acids. The strongest negative correlations were shown by another enzyme of nucleotide metabolism – nicotinamide riboside kinase, and also a gene with a presently unknown function – *PQ loop repeat-containing protein*.

**Figure 5:**
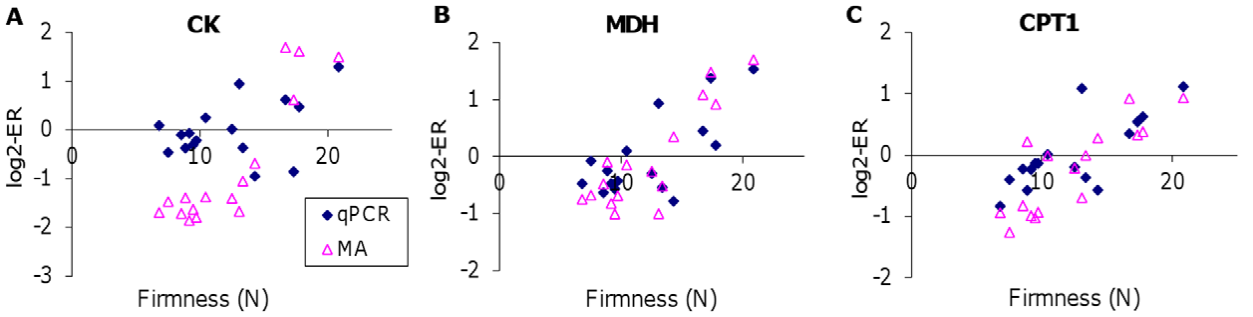
Relationship between fillet firmness and A: creatine kinase (CK), B: malate dehydrogenase (MDH), and C: carnitine O-palmitoyltransferase I (CPT1) determined with microarray (MA) or by real time quantitative RT-PCR (qPCR).

Three genes selected by the microarray results were validated with qPCR and association of their expression with firmness of fillets was confirmed (Fig. 5). Microarray analyses did not find differential expression of *myogenin*, *PPARα* or *PPARβ*. However, these genes were included in the qPCR analyses based on their important roles in development and functions of skeletal muscle [31]–[33]. Neither of these three genes showed strong correlations with the studied trait (r < |0.55|), data not shown).

## Discussion

The firmness of the salmon fillets in the current work covered the whole range from soft to very firm muscle, with an average value corresponding to normal texture of commercially reared Norwegian salmon (8–11 N) [16]. It is important to know the heritability of a trait for planning and implementing a selective breeding program. Also, heritability is important for predicting both the response to selection and individual breeding values [34]. Texture can be included as a goal in breeding programs, but the low to moderate heritability indicates a slow improvement in texture in progeny from selected parents. As some quality traits such as texture necessarily require the sacrifice of the individual, it is not possible to measure these traits on breeding candidates themselves, rather, closely-related relatives (usually siblings) are used for this purpose [35]. This is more difficult for traits of low and medium heritability, thus development of methods to test breeding candidates themselves can be highly beneficial. Gene expression can be analyzed in biopsy samples with no significant damage to the fish, thus enabling the prediction of high fillet quality in live salmon which will assist selective breeding programs.

Despite the importance of fillet firmness for commercial Atlantic salmon aquaculture, there is only limited knowledge about molecular features associated with this trait. Among multiple possibilities, one may anticipate a relationship between firmness and characteristics of skeletal muscle, including composition, morphology, structure of the ECM (contents of proteins and glycans, cross-linking of collagens), proteolysis, inflammation, cell death and oxidative stress. Microarray analyses are well suited for work with poorly investigated traits and conditions. Overall, multiple gene expression does not require any prior assumption or hypotheses. An important advantage of microarray over RNA sequencing, a competing approach to transcriptomic profiling is that identification and annotation of genes are performed before analyses. Our oligonucleotide platforms for several aquaculture species were designed with an aid of bioinformatic system (STARS), which is also used as a knowledge base and includes several utilities for data analyses. This facilitates and enhances interpretation of results. Analyses of microarray data commonly begin with selection of genes, which in this study was based on correlation with firmness of salmon fillets. Search for enriched terms helps to identify thematic associations of the gene lists. This simple approach is useful at an initial stage of data analyses. However, it is necessary to keep in mind its limitations. Annotations of genes in public databases are far from being exact and complete. Custom annotations performed in STARS found many functional associations that were not represented in GO or KEGG. Enrichment analysis and other operations with GO categories and pathways presume that functionally related genes have similar expression profiles, which does not hold true in many cases. GO categories include genes with related roles that change expression under different conditions and therefore co-regulation is not always observed. Hence, mining of microarray data is dealing with both groups and individual genes. Given high heterogeneity of GO categories, performance of groups found by an enrichment analysis requires careful inspection.

A specific feature of this study was high correlation of expression profiles in several functional groups. To assess relationship between gene expression and fillet quality, regression analyses were performed using the mean log_2_-ER values. Averaging of data reduced random fluctuations and revealed the general trend, namely strong association with firmness. Most of these functional groups were related to metabolism. Results suggested that in the analyzed salmon, firmness depended mainly on intracellular processes. The list of DEG does not include genes that control differentiation of skeletal muscle and qPCR analyses did not find association of the myogenic regulator myogenin with firmness.

Softness of flesh was most likely not associated with cell death or inflammation. Genes involved in apoptosis did not show differential expression. A small number of immune genes tended to have higher transcription in firm fillets and this group did not include genes that are typically seen in the signatures of inflammation (e.g. cytokines, chemokines and their receptors, lectins, antibacterial proteins and complement components, proteins implicated in oxidative burst, matrix metalloproteinases and other effectors). Microarray analyses commonly find up-regulation of these genes under inflammatory conditions. In earlier studies we observed differential expression of genes involved in various processes taking place in mitochondria [36], but this study was unique in the scale of differences that encompass virtually all mitochondrial functions. This is likely due to massive propagation of mitochondria when all components need to be produced simultaneously. Expression profiles of mitochondrial genes strongly suggest association of firmness with high rates of aerobic metabolism that uses fat as a main fuel. This was confirmed with expression of genes involved in lipid metabolism and biogenesis of peroxisomes. On the contrary, individuals with soft fillets tended to have higher levels of anaerobic metabolism, while sugar metabolism separated salmon with firm fillets into a distinct group (upper 20^th^ persentile). Lower expression of myofiber proteins could be a consequence of co-regulation with genes involved in glycolysis, a major source of energy in white skeletal muscle. Expression profiles of proteasome components and lysosomal proteases suggested higher rate of protein degradation in fillets with high firmness. This may seem unexpected and counter-intuitive since high activity of proteolytic enzymes, such as cathepsins [7], [14], [37] and collagenases [14], [38] has been associated with soft flesh. It is possible that damage is produced by uncontrolled protein degradation while rapid removal of abnormal proteins may be important for maintenance of tissues in a good condition. High integrity of muscle tissue has been associated with increased deposition of ECM [39]–[41]. However, a number of ECM-related genes were up-regulated in soft fish in the current work. Strong correlation with fillet firmness was found for individual genes from all mentioned groups and also for a number of genes with unknown functions or those whose associations with the trait were hard to interpret. These genes can be regarded as candidate markers of fillet quality of Atlantic salmon.

This study took advantage of highly standardized fish material from a large breeding program. This reduced interference of side factors that may affect fillet quality and obscure its dependence on gene expression. Firmness was determined predominantly with inherent properties of fish and this made it possible to find strong relationships between this trait and a suite of genes and functional groups. Despite the novel results in this study, it is important to consider that fillet quality characteristics such as texture may be related to different factors. It is, as-yet, undetermined whether gene expression differences between salmon with soft and firm flesh developed within a long time period or appeared rapidly during transportation from farm to slaughter. We also do not know whether the metabolic properties of skeletal muscle were inherited or rather if they appeared in some fish due to uneven distribution in the cages and exposure to hypoxic conditions. Answers to these questions are important in order to enable improvement of salmon fillet texture. If a higher rate of anaerobic metabolism and concomitant reduction of quality develop in individuals due to low oxygen levels, correction may be achieved by environmental improvement. In the case that metabolic differences are inherited, breeding will likely be a more effective strategy.

## Supporting Information

Table S1 Single genes from microarray analysis that passed quality control.
(XLS)

Table S2 Differentially expressed genes that passed quality control.
(XLS)

Table S3 Gene composition of functional groups showing strong association (|r| >0.7) with fillet firmness.
(XLS)
